# COVID-19 Pandemic Experiences in Pediatric Intensive Care Unit: An Iranian Referral Hospital-Based Study

**DOI:** 10.1155/2022/1682986

**Published:** 2022-10-25

**Authors:** Masoud Mohammadpour, Seyed Abbas Hassani, Meisam Sharifzadeh, Leila Tahernia, Setareh Mamishi, Bahareh Yaghmaie, Zeinab Najafi, Farzaneh Beirami, Mehrnoush Afsharipour, Maryam Minuyeefar, Mina Dolatzadeh, Neda Pak, Anahita Majmaa, Zahra Zamani, Shima Mahmoudi

**Affiliations:** ^1^Division of Pediatric Intensive Care Unit, Pediatrics Center of Excellence, Children's Medical Center, Tehran University of Medical Sciences, Tehran, Iran; ^2^Department of Infectious Diseases, Pediatrics Center of Excellence, Children's Medical Center, Tehran University of Medical Sciences, Tehran, Iran; ^3^Pediatric Infectious Disease Research Center, Tehran University of Medical Science, Tehran, Iran; ^4^Pediatrics Center of Excellence, Children's Medical Center, Tehran University of Medical Sciences, Tehran, Iran

## Abstract

**Introduction:**

In late February 2020, after we had informed about the presence of some cases of COVID-19 in Iran and its rapid spread throughout the country, we decided to make the necessary arrangements for patients with critical conditions in Pediatric Intensive Care Unit (PICU) at Children's Medical Center. There are a little data on critically ill children with COVID-19 infection with ICU requirements. The aim of this study was to describe clinical characteristics, laboratory parameters, treatment, and outcomes of the pediatrics population infected by SARS-CoV-2 admitted to PICU.

**Materials and Methods:**

This study was performed between February 2020 and May 2020 in the COVID PICU of the Children's Medical Center Hospital in Tehran, Iran. Patients were evaluated in terms of demographic categories, primary symptoms and signs at presentation, underlying disease, SARS-CoV-2 RT-PCR test result, laboratory findings at PICU admission, chest X-ray (CXR) and lung CT findings, and treatment. Moreover, the need to noninvasive ventilation (NIV) or mechanical ventilation, the length of hospital stay in the PICU, and outcomes were assessed.

**Results:**

In total, 99 patients were admitted to COVID PICU, 42.4% (42 patients) were males, and 66 patients had positive SARS-CoV-2 real-time reverse transcriptase-polymerase chain reaction (RT-PCR). There was no statistically significant difference in the frequency of clinical signs and symptoms (except for fever) among patients with positive SARS-CoV-2 RT-PCR and negative ones. Among all admitted patients, the presence of underlying diseases was noticed in 81 (82%) patients. Of 99 patients, 34 patients were treated with NIV during their admission. Furthermore, 35 patients were intubated and treated with mechanical ventilation. Unfortunately, 11 out of 35 mechanically ventilated patients (31%) passed away.

**Conclusion:**

No laboratory and radiological findings in children infected with COVID-19 were diagnostic in cases with COVID-19 admitted to PICU. There are higher risks of severe COVID-19, PICU admission, and mortality in children with comorbidities.

## 1. Introduction

In December 2019, several cases with severe pneumonia were admitted to hospitals in the Wuhan city of China. It was gradually revealed that they are caused by a novel coronavirus named severe acute respiratory syndrome coronavirus 2 (SARS-CoV-2) and spread around the world [1].

The diagnosis of the coronavirus disease 2019 (COVID-19) is based on detection of SARS-CoV-2 in nasopharyngeal swab samples using reverse transcription polymerase chain reaction (RT-PCR) [2, 3]. Based on the experience of Chinese physicians, a computerized tomography (CT) scan has a sensitivity of 97% for the diagnosis of COVID-19, which is higher than the RT-PCR test of the pharyngeal specimen [4]. According to the previous reports, all age groups of humans are vulnerable to coronavirus, but some groups including elderlies and those with underlying chronic conditions are more likely to develop into a critical condition. The presentation of disease in children with COVID-19 is not usually severe along with the least mortality [5]. Based on recent reports, pediatric cases with COVID-19 are usually ranging from 1.5 months to 17 years of age [4]. In severe cases of COVID-19 in adult patients, dyspnea impeding to acute respiratory distress syndrome (ARDS), coagulopathy, severe metabolic acidosis refractory to treatment, and septic shock were reported frequently [6]; however, in children despite mild symptoms as a dominant presentation of COVID-19, death might be considered a consequence of this virus especially in those with underlying chronic disease.

According to previous reports, a spectrum of SARS-CoV-2 infection might occur in children; however, a milder clinical course of disease appears in most infected children [3, 6–10]. Moreover, many children and adolescents requiring intensive care have been hospitalized with multisystem inflammatory symptoms, Kawasaki-like symptoms, and toxic shock syndrome [11–14]. On the basis of data published by Chinese articles, it was not expected initially to encounter with lots of pediatric cases of COVID-19; however, since April 13, 2020, we were obliged to devote a complete Pediatric Intensive Care Unit (PICU) setting to a high number of patients requiring critical care. The total patients presented in this paper include those who have been admitted to COVID PICU at Children's Medical Center, an Iranian referral pediatrics hospital, from late February to 21^st^ of May, 2020, with the initial diagnosis of COVID-19 based on presenting clinical and radiological symptoms and laboratory parameters in favour of COVID-19.

In late February 2020, after we have informed about the presence of some cases of COVID-19 in Iran and its rapid spread throughout the country, we decided to make the necessary arrangements for patients with critical conditions in PICU at Children's Medical Center.

Although intensive care service plays a very important part of healthcare in both developing and developed countries [15], there are a little data on critically ill children with COVID-19 infection with ICU requirements. The aim of this study was to describe clinical characteristics, laboratory parameters, treatment, and outcomes of the pediatrics population infected by SARS-CoV-2 admitted to PICU.

## 2. Materials and Methods

This study was performed between February 2020 and May 2020 in the COVID PICU of Children's Medical Center Hospital in Tehran, Iran. The COVID PICU (level 2), a medical and cardiac intensive care, is a 10 bed unit. Nearly 700 critically ill patients are admitted in the PICU each year for specialized medical and nursing services.

### 2.1. Definitions

Based on a positive SARS-CoV-2 real-time reverse transcriptase-polymerase chain reaction (RT-PCR) test [2] and/or strong clinical manifestations, radiological or laboratory findings were compatible with this disease, and our patients were included as COVID-19 cases.

### 2.2. Ethics

The protocol of this retrospective study was approved by the Ethics Committee of Tehran University of Medical Sciences and Children's Medical Center Hospital.

### 2.3. Data Collection

This study is a retrospective cross-sectional study. Inclusion criteria were defined as follows: cases of COVID-19 proved by a positive SARS-CoV-2 RT-PCR test or cases that were diagnosed by strong clinical, radiological, or laboratory findings.

Exclusion criteria included patients who left the COVID PICU for some reason after admission and did not undergo any initial diagnostic or therapeutic measures.

### 2.4. Protocols and Team

The diagnostic and therapeutic protocols used for these patients were based on the latest NIH and WHO guideline as well as the COVID-19 Diagnostic and Therapeutic Guideline of the Ministry of Health of the Islamic Republic of Iran.

The treatment team included the pediatric ICU fellow, pediatric infectious disease specialist, pediatric cardiologist, pediatric pulmonologist, pediatric rheumatologist, and pediatric radiologist.

Patients were evaluated in terms of demographic categories, primary symptoms and signs at presentation, underlying disease, SARS-CoV-2 RT-PCR test result, laboratory findings at PICU admission, chest X-ray (CXR) and lung CT findings, and treatment. Moreover, the need to NIV or mechanical ventilation, the length of hospital stay in the PICU, and outcomes were assessed.

A sample of nasal swabs was taken for SARS-CoV-2 RT-PCR at the beginning of patients' admission to the PICU. CXR and lung CT scan findings were interpreted by a pediatric radiologist.

Information recorded included demographic data, underlying comorbidities, clinical features, laboratory findings and radiologic assessments, severity of disease, necessity of intubation and ventilation, and mortality. All pieces of information were extracted from the clinical files and records, nursing reports, laboratory, and radiology reports. The respiratory distress at the time of PICU admission was classified according to clinical respiratory scores (CRS) into three categories, severe, moderate, and mild.

### 2.5. Statistical Analysis

Finally, the results of reviewing the files of 99 patients, which were summarized in the questionnaires, were uploaded in Excel software and statistically analysed using SPSS software (Statistical Package for the Social Sciences) version 13.0 software (SPSS Inc).

Categorical variables were described as frequency rates and percentages, and continuous variables were described using median and interquartile range (IQR) values. Normally distributed continuous variables were presented as means with standard deviations (SD). The chi-square test was the main test used for statistical analysis between the two groups. Variables with a two-tailed *P* value < 0.05 were considered statistically significant.

## 3. Results

In total, 99 patients were admitted to COVID PICU, the mean age of the patients was 5.9 ± 5.1 years, and 42.4% (42 patients) were males. In 66.7% (66 patients), SARS-CoV-2 RT-PCR tests were positive. In patients with positive SARS-CoV-2 RT-PCR, 28 patients were males and 38 patients were females (Table 1). In terms of age distribution, in patients with positive SARS-CoV-2 RT-PCR, the most prevalent ones were reported in patients aged less than one-year olds (16 patients) and 3 to 4-year olds (6 patients).

Among 99 admitted patients, the presence of underlying diseases was noticed in 81 (82%) patients. Among these patients, 34 were treated with noninvasive ventilation (NIV) during their admission. Furthermore, 35 patients were intubated and treated with mechanical ventilation. Among 66 patients with positive SARS-CoV-2 RT-PCR, 53 patients (80%) were suffering from a known underlying disease. The most prevalent underlying diseases in this group were as follows: congenital heart disease (10 patients, 15%), chronic lung disease (9 patients, 14%), chronic neurological disorder (7 patients, 11%), leukaemia and solid tumours (6 patients), IEM and graft-versus-host disease (GvHD) following bone marrow transplantation with five patients (8%) in each group and the primary immune deficiency (3 patients, 5%) (Table 1). Twenty-one out of 66 patients with positive SARS-CoV-2 RT-PCR and 14 out of 33 patients with negative SARS-CoV-2 RT-PCR were intubated and treated with mechanical ventilation. Continuous renal replacement therapy (CRRT) was applied in 1 patient, and therapeutic plasma exchange, high-frequency oscillatory ventilation (HFOV), and extracorporeal membrane oxygenation (ECMO) were not used for any cases. Unfortunately, 11 out of 35 mechanically ventilated patients (31%) passed away.

According to the statistical results, the necessity of mechanical ventilation in patients (with positive and negative SARS-CoV-2 RT-PCR) with the primary diagnosis of COVID-19 could deteriorate the prognosis. NIV was used in 25 patients with positive SARS-CoV-2 RT-PCR (38%). In total, 8 and 3 patients passed away in each group, respectively. Administration of noninvasive ventilation in patients with positive SARS-CoV-2 RT-PCR admitted in PICU could not be considered a mortality indicator. Moreover, 37.5% of positive SARS-CoV-2 RT-PCR who were admitted in PICU and discharged in a good condition have been intubated and treated with mechanical ventilation during their admission. While in a dead patient with positive SARS-CoV-2 RT-PCR, this accounts for 100% according to the statistics. Intubation and mechanical ventilation in patients with COVID-19 result in poor prognosis (*P* value = 0.002).

However, there is no significant difference in prognostic evaluation in patients with negative SARS-CoV-2 RT-PCR between the two groups undergoing NIV or mechanical ventilation and those who were not treated with NIV or mechanical ventilation.

There was no statistically significant difference in the frequency of clinical signs and symptoms among patients with positive SARS-CoV-2 RT-PCR and negative ones. The only exception was detected in the frequency of fever that was statistically significant between the two groups of patients with a negative and positive SARS-CoV-2 RT-PCR test (*P* value: 0.030). In patients with a positive SARS-CoV-2 RT-PCR test, fever was the most prevalent symptom (53 patients, 80.3%). The frequency of other important symptoms were as follows: respiratory distress in 62.1% (41 patients), tachypnea in 59.1% (39 patients), cough in 51.5% (34 patients), malaise in 36.4% (24 patients), vomiting in 34.8% (23 patients), cyanosis and abdominal pain in 21.2%, decreased level of consciousness in 19.7(13 patients), diarrhea, shock state signs, headache, and gastrointestinal bleeding were detected in 16.7%, 15.2%, 20.1%, and 10.6%, respectively. Among 33 patients with negative SARS-CoV-2 RT-PCR, 22 patients (66.7%) had no respiratory involvement in favour of COVID-19 which was detected in the spiral lung CT scan. However, in 66 patients with positive SARS-CoV-2 RT-PCR, lung involvement in support of COVID-19 was reported in 33.3% (22 patients). On the other hand, 33.3% (11 patients) with negative SARS-CoV-2 RT-PCR showed lung involvement. According to this data, no significant difference was noted with regard to radiological presentation of COVID-19 in the spiral lung CT scan in two groups.

The most prevalent respiratory presentation of COVID-19 on CT scans of admitted patients in PICU with positive SARS-CoV-2 RT-PCR included bilateral patchy shadowing in 36.4% (24 patients), patchy infiltration in 30.3% (20 patients), consolidation in 22.7% (15 patients), subpleural lesion (13.6%), ground glass opacity (12.1%), and pleural effusion (10.6%). Local patchy shadowing, lymphadenopathy, interstitial abnormality, crazy paving halo sign, tree in bud, peribronchial thickening, and pericardial effusion were discovered in 1.5% to 9.1% of patients. The prevalence of radiological presentations was evaluated in PICU admitted patients with negative SARS-CoV-2 RT-PCR. Considerable statistical difference was reported in common patterns of respiratory involvements in spiral CT scans. It can be detected that in hospitalized patients with clinical symptoms suspected to COVID-19 (in both groups with positive and negative tests), no significant difference was detected in spiral CT scan reports.

### 3.1. The Relation between Respiratory Distress and Mortality

All dead patients suffered from moderate to severe respiratory distress at the time of admission in PICU, while other 14 patients were admitted in PICU without respiratory distress.

In total, 13, 33, and 28 patients had mild, moderate, and severe respiratory distress, respectively (Table 2). Besides, considerable relation was discovered between respiratory distress and increase in mortality. The relation between severity of respiratory distress at the time of admission and mortality was significant (*P* value = 0.034).

### 3.2. The Relation between Underlying Disease and Mortality

All dead patients suffered from an underlying disease (3 patients) with negative SARS-CoV-2 RT-PCR and 8 patients with positive SARS-CoV-2 RT-PCR. Of the 88 patients who were discharged from PICU, 67 patients (77%) had underlying diseases. Moreover, 45 out of 66 patients with positive SARS-CoV-2 RT-PCR and underlying disease were discharged from PICU, and 8 patients died. Thirteen patients with positive SARS-CoV-2 RT-PCR and underlying disease were discharged from PICU as well. On the other hand, it could not be concluded that the presence of a positive SARS-CoV-2 RT-PCR test in a patient with an underlying disease would result in an increase in mortality. The most prevalent causes of mortality in admitted patients in PICU with positive SARS-CoV-2 RT-PCR were leukaemia and solid tumor (3 patients), chronic respiratory diseases including asthma and cystic fibrosis (3 patients), bone marrow transplantation (2 patients), methylmalonic acidemia (MMA)-2 IEM (1 patient), primary immune deficiency (Mendelian susceptibility to mycobacterial disease) (1 patient), congenital heart disease (1 patient), and fulminant hepatic failure (1 patient).

Although in this study, all dead patients had an underlying disease, a statistically significant relationship was not found between positive SARS-CoV-2 RT-PCR and negative SARS-CoV-2 RT-PCR patients (*P* value = 0265).

### 3.3. The Duration of Admission, Intubation, and Mechanical Ventilation

Duration of admission in PICU of SARS-CoV-2 RT-PCR positive patients was approximately 4 days, which was not statistically different from cases with negative SARS-CoV-2 RT-PCR (*P* value = 0.65).

### 3.4. The Vital Signs at the Time of Admission in PICU

The oxygen saturation in 41 out of 66 patients with positive SARS-CoV-2 RT-PCR was less than 94%. This saturation was detected in 17 out of 33 patients with negative SARS-CoV-2 RT-PCR which was not statistically noticeable as well (*P* value > 0.05).

The most considerable sign in majority of patients was tachycardia at the time admission in PICU (50 patients with positive SARS-CoV-2 RT-PCR and 22 patients with negative SARS-CoV-2 RT-PCR (*P* value > 0.05).

Tachypnea was another frequent sign in these patients. Forty-five out of 66 patients (positive SARS-CoV-2 RT-PCR) and 24 out of 33 patients (negative SARS-CoV-2 RT-PCR) were tachypneic at the time of admission (*P* value > 0.05). Hypertension was detected in 14 out of 66 patients with positive SARS-CoV-2 RT-PCR (*P* value > 0.05).

### 3.5. The Laboratory Findings in Patients with Positive and Negative SARS-CoV-2 RT-PCR

Table 2 shows laboratory findings on admission. No significant difference was found in laboratory tests (except for ferritin and PCO2) of the patients with positive and negative SARS-CoV-2 RT-PCR (Table 3).

## 4. Discussion

Since December 2019, an outbreak of coronavirus disease 2019 (COVID-19) has spread globally. Although the increasing number of documented cases of SARS-CoV-2 has been reported, little is known about the epidemiological and clinical features of pediatric patients with COVID-19 with severe disease who require ICU admission.

In this study, the epidemiological, laboratory, and clinical features of 99 pediatric patients with severe acute respiratory symptoms admitted to PICU of an Iranian referral hospital were analysed. One-third of COVID-19 patients admitted to PICU had negative SARS-CoV-2 RT-PCR tests that might be due to the low sensitivity (32% to 63%) of pharyngeal and nasal swab [16]. According to the COVID-NET reports, although the cumulative COVID-19 hospitalization rates for children less than 18 years were much lower than adults (8/100,000 population vs. 164.5/100,000), the need of ICU admission among hospitalized children was similar to adults (33.2% vs. 32%) [17].

According to the study of Rajmil et al., between 15% and 55%–60% of children with COVID-19 are asymptomatic, and family transmission can occur in 75%–100% of the cases. Surprisingly, children are not COVID-19 transmitters greater in extent than adults [18]. The case-fatality rate in China reported recently as 2%, and mortality rate is increasing considerably in older people (around 15%). According to some studies from China, children aged younger than 10 years account for only 1% of COVID-19 cases. However, infants and young children are typically at high risk for hospitalization after respiratory tract infection. Immaturity of the respiratory tract and immune system was an important contributor to severe viral respiratory disease in children [19].

In total, 13, 33, and 28 patients had mild, moderate, and severe respiratory distress, respectively. In our study, 82% of the patients had underlying diseases. According to previous meta-analysis, there is a higher risk of severe COVID-19 and ICU admission in children with comorbidities (relative risk ratio of 1.79); however, the effects of underlying diseases on COVID-19 severity in children are still unclear [20].

Among the 99 patients admitted to PICU, 35% were intubated and treated with mechanical ventilation, and 31% of them (*n* = 11) died. A past medical history of an underlying condition is considered the main risk factor for death in children with COVID-19 [21]. All dead patients showed moderate to severe respiratory distress at admission in PICU, and a significant relation between respiratory distress and increase in mortality was found.

According to data from China, pediatric cases with COVID-19 might show less severe disease than adults, and children might experience different symptoms than adults [6, 22]).

In our study, no significant differences were found in the frequency of clinical signs and symptoms (except for fever) among patients with positive SARS-CoV-2 RT-PCR and negative RT-PCR.

Children usually present with gastrointestinal symptoms in comparison with adults. Most children frequently present COVID-19 with fever, but recently, this is not usually found in cases with COVID-19 caused by CoVs with novel mutations [23].

In this study, no significant differences in laboratory tests (except for ferritin and PCO2) were found in cases with positive and negative SARS-CoV-2 RT-PCR.

It has been reported that hypoxia and acidosis might lead to the development of severe COVID-19 [24]. In our study, acidosis (decreased PH) was found in 42.4% and 21.2% of cases with negative and positive SARS-CoV-2 RT-PCR, respectively.

This study has several limitations. First, since our hospital is a referral pediatrics center and most of the children with complicated conditions are referred, our results might show a more serious type of COVID‐19; therefore, these results should be interpreted with caution. Second, due to the low sensitivity of RT-PCR and its limitation, we considered all cases of COVID-19 proved by the positive SARS-CoV-2 RT-PCR test or those which were diagnosed by strong clinical, radiological, or laboratory findings as COVID-19.

In conclusion, no laboratory and radiological findings in children infected with COVID-19 were diagnostic in cases with COVID-19 admitted to PICU. There are higher risks of severe COVID-19, PICU admission, and mortality in children with comorbidities.

## Figures and Tables

**Table 1:** The demographic data of 66 patients with positive SARS-CoV-2 RT-PCR.

Variables	
Age (Mean ± SD, year)	6.1 ± 5.2

Sex (male, N (%))	28 (42%)
*Underlying conditions, N (%)*	
Congenital heart disease	10 (15)
Chronic lung diseases	9 (14)
Chronic neurological disorder	7 (11)
Leukaemia and solid tumors	6 (9)
Graft-versus-host disease (GvHD) following bone marrow transplantation	5 (8)
Inborn errors of metabolism (IEM)	5 (8)
Primary immune deficiency	3 (5)
Liver diseases	2 (3)
Neuromuscular disorder	2 (3)
Down syndrome	1 (1.5)
Multiple trauma	1 (1.5)
Epidermolysis bullosa	1 (1.5)
Systemic lupus erythematosus	1 (1.5)

Treatment, N (%)	
Noninvasive ventilation (NIV)	25 (38)
Invasive mechanical ventilator (IMV)	21 (32)

**Table 2:** The clinical respiratory score at the time of admission to the ICU.

Group	No respiratory distress, N (%)	Mild respiratory distress, N (%)	Moderate respiratory distress, N (%)	Severe respiratory distress, N (%)	Total (N)
Survived patients	14 (16)	13 (15)	33 (37.5)	28 (32)	88
Nonsurvived patients	0	0	5 (45)	6 (56)	11
Total	14 (14)	13 (13)	38 (39)	34 (34)	99

**Table 3:** Laboratory findings of the cases with positive and negative SARS-CoV-2 RT-PCR.

Laboratory findings	Negative SARS-CoV-2 RT-PCR (*n* = 33)	Positive SARS-CoV-2 RT-PCR (*n* = 66)	*p* value
Leukopenia	3 (9)	14 (21.2)	0.22
Leucocytosis	19 (57.6)	27 (40.9)	
Anaemia	9 (27.3)	13 (19.7)	0.26
Thrombocytopenia	6 (18.2)	18 (27.3)	0.45
Thrombocytosis	6 (18.2)	8 (12.1)	
Lymphopenia	17 (51.5)	26 (39.4)	0.66
Increased ESR	24 (72.7)	48 (72.7)	1
Increased CRP	20 (60.6)	48 (72.7)	0.23
Increased SGOT	8 (24.2)	17 (25.7)	0.69
Increased SGPT	8 (24.2)	14 (21.2)	0.88
Decreased albumin	15 (45.5)	29 (43.9)	0.5
Increased LDH	8 (24.2)	19 (27.8)	0.58
Increased CPK	4 (12.1)	9 (13.6)	0.57
Increased CK-MB	7 (21.2)	18 (27.3)	0.19
Increased troponin-I	1 (3)	2 (3)	1
Increased ferritin	11 (33.3)	34 (51.5)	0.03
Increased IL6	4 (12.1)	13 (19.7)	0.19
Decreased fibrinogen	11 (33.3)	10 (15.1)	0.055
Increased D-dimer	7 (21.2)	6 (9)	0.31
Hyponatremia	18 (54.5)	29 (43.9)	0.35
Hypokalaemia	10 (30.3)	21 (31.8)	0.84
Hypocalcaemia	18 (54.5)	28 (42.4)	0.34
Hypomagnesaemia	5 (15.1)	5 (7.6)	0.21
Increased BUN	6 (18.2)	11 (16.7)	0.57
Increased creatinine	5 (15.1)	12 (18.2)	0.68
Acidosis (decreased bicarbonate)	20 (60.6)	43 (65.1)	0.39
Acidosis (decreased PH)	14 (42.4)	14 (21.2)	0.09
Decreased PCO2	19 (57.6)	41 (62.1)	0.018
Increased PCO2	10 (30.3)	6 (9)	
Decreased saturation (less than 94%)	20 (60.6)	30 (45.4)	0.2
Increased PT	23 (69.7)	41 (62.1)	0.92
Increased PTT	7 (21.2)	13 (19.7)	0.89

ESR : erythrocyte sedimentation rate, CRP : C-reactive protein, SGOT : serum glutamic-oxaloacetic transaminase, SGPT : serum glutamic pyruvic transaminase, LDH : lactate dehydrogenase, CPK : creatine phosphokinase, CK-MB : creatine kinase-MB, BUN : blood urea nitrogen, PT : prothrombin time, PTT : partial thromboplastin time.

## Data Availability

The data used to support the findings of this study are available from the corresponding author upon request.
